# The Therapy of Pulmonary Fibrosis in Paracoccidioidomycosis: What Are the New Experimental Approaches?

**DOI:** 10.3390/jof6040217

**Published:** 2020-10-11

**Authors:** Ángel González

**Affiliations:** Basic and Applied Microbiology Research Group (MICROBA), School of Microbiology, Universidad de Antioquia, 050031 Medellin, Colombia; angel.gonzalez@udea.edu.co; Tel.: +57-(4)-219-5489; Fax: +57-(4)-219-8494

**Keywords:** *Paracoccidioides* spp., paracoccidioidomycosis, pulmonary fibrosis, therapy, treatment, itraconazole

## Abstract

Pulmonary fibrosis (PF) is considered the most important sequela developed in patients suffering from the chronic form of paracoccidioidomycosis (PCM), which leads to the loss of respiratory function in 50% of cases; this residual pulmonary abnormality is present even after antifungal treatment. To date, there is no effective treatment for PF. However, the use of antifungal drugs in combination with other antibiotics or immunomodulatory compounds, as well as biological therapies that include a monoclonal antibody specific to neutrophils, or prophylactic vaccination employing a recombinant antigen of *Paracoccidioides brasiliensis* that successfully attenuated PF, has been reported. Additionally, mesenchymal stem cell transplantation in combination with antifungal therapy slightly reduced the inflammatory response and profibrotic molecules induced by *P. brasiliensis* infection. In this review, I report experimental findings from several studies aiming to identify promising therapeutic strategies for treating PF developed in PCM.

## 1. Introduction

Pulmonary fibrosis (PF) is a progressive lung disease that develops as a result of a repetitive injury to the alveolar tissue, mainly the alveolar epithelium, which triggers the immune system to restore the tissue architecture of the damaged tissue. In this process, several inflammatory mediators, including cytokines and extracellular matrix (ECM) components, which if produced abnormally, lead to an excess of ECM deposition and the subsequent scarring of the tissue [1].

Idiopathic pulmonary fibrosis (IPF), whose cause is unknown, is the most common type of PF, as well as the most severe form of interstitial lung diseases [2]. Conversely, PF is produced by long-term exposure to several factors including minerals such as asbestos, silica, and coal dust; physical factors such as radiation, and some chemotherapy agents (bleomycin, methotrexate) and anti-inflammatory compounds (rituximab, sulfasalazine), as well as heart medications (propranolol, amiodarone), antibiotics (ethambutol, nitrofurantoin), and microbial pathogens [2,3]. Several studies have demonstrated that viruses (Epstein-Barr virus (EBV), cytomegalovirus (CMV), and certain types of Herpes simplex virus (HSV)), bacteria (*Streptococcus pneumoniae* and *Mycobacterium tuberculosis*), and fungi (*Paracoccidioides brasiliensis* and *Aspergillus fumigatus*) are also involved in the development of PF [4].

In the case of paracoccidioidomycosis (PCM), a systemic and endemic mycosis is restricted to Latin America and caused by dimorphic fungal pathogens belonging to the genus *Paracoccidioides*, approximately 60% of patients suffering the chronic form of the mycosis develop PF [5]. Apparently, this sequela is due to persistent fungal antigen stimulations and the subsequent activation of the immune system with alteration to its repair mechanisms [6]. Notwithstanding the effective use of antifungal therapy for long periods, for the treatment of active PCM, does not appear to affect the development of PF [7].

Currently, there is no effective therapy for treating PF developed in patients with PCM. Nonetheless, in the last years, some experimental strategies employing a pulmonary fibrosis model induced by *P. brasiliensis* infection have been shown to be effective in reducing PF [8,9,10,11,12]. In this review, I summarize the different experimental therapeutic approaches that have been developed so far to treat pulmonary fibrosis in PCM. I provide evidence that the combination of antifungal drugs with immunomodulatory compounds, antibiotics, as well as with biological therapies based on the use of a monoclonal antibody specific to neutrophils, mesenchymal stem cell transplantation, or vaccination with a specific antigen of *P. brasiliensis*, successfully reduced not only the granulomatous inflammatory response but also attenuated the PF.

## 2. Paracoccidioidomycosis and Development of Pulmonary Fibrosis

Paracoccidioidomycosis is considered one of the most important systemic and endemic mycoses. This mycosis is restricted to Latin America and is caused by the dimorphic fungal pathogen from the genus *Paracoccidioides* [13]. It is estimated that 10 million people are infected with this pathogen, of which only about 1–2% will develop the mycosis [14,15]; however, among the chronic fungal diseases, PCM exhibits one of the highest mortality rates with approximately 51% of the deaths in Brazil [16], and with an incidence of 2.7 new cases per 100.000 habitants per year [17]. The clinical presentation of this mycosis covers the acute and the chronic form, with the latter representing about 90% of the cases [18]. The lung is the primary site of infection with the development of lesions that may progress to a granulomatous inflammatory response with tissue damage [19,20]. This progressive chronic inflammation is associated with a persistent fungal antigen stimulation that leads to the development of PF in at least 60% of the patients suffering from the chronic form of this mycosis [7,21].

Experimental models of pulmonary PCM that imitate the natural occurrence in human patients have allowed us to understand the course of the disease. In these models, it has been observed that the granulomatous and fibrogenic processes begin at four-week post-infection and are established and consolidated from an eight-week post-challenge [10,22,23]. PF development is associated with granulomatous inflammation and leukocyte infiltration, mainly neutrophils, eosinophils, mononuclear cells (subpopulations of both macrophages and lymphocytes), myeloid derived-suppressor cells (MDSCs), and fibrocytes (all cells enumerated by flow cytometry), followed by an increase of pro-inflammatory and pro-fibrotic cytokine production including tumor necrosis factor alfa (TNF-α), transforming growth factor-beta (TGF-β), interleukin (IL)-1, IL-6, IL-13, and IL-17 [10,24]. Moreover, an exaggerated deposition of ECM proteins, mainly collagen and reticulin, and the increased production of hydroxyproline are also noticed, as a consequence of the activation of fibroblasts by pro-fibrotic cytokines produced in turn by activated-macrophages [24,25]. Moreover, this pathological condition is also accompanied by the production and over-activation of proteolytic enzymes such as metalloproteinase (MMP)-8, responsible for ECM remodeling and degradation, as well as by an increase of tissue inhibitor metalloproteinase (TIMP)-2 in an attempt to neutralize the MMP and to prevent excessive ECM proteins degradation [10].

**Figure 1:**
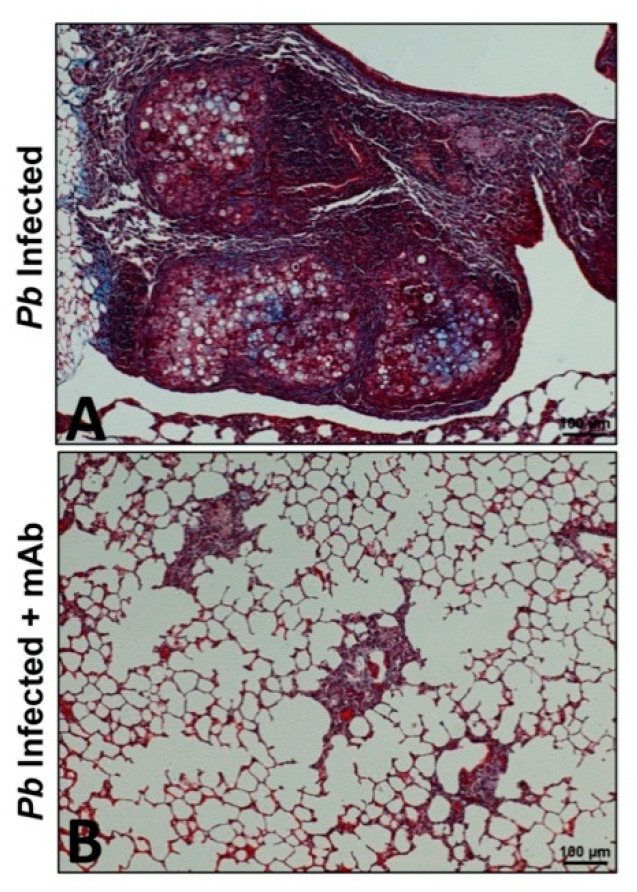
A monoclonal antibody against neutrophils-reduced pulmonary fibrosis in the lungs of mice infected with *P. brasiliensis*. Microphotographs are representative of lungs from infected and untreated mice (**A**) or infected and treated with a monoclonal antibody (anti-Ly6G) specific to neutrophils (**B**) at 12 weeks post-challenge. The lungs were fixed, embedded in paraffin, cut, and stained using Masson’s trichrome to identify and differentiate collagen fibers. Magnification 10×.

At the histopathological level, the architecture of the lung parenchyma shows a granulomatous cellular infiltrate composed mainly of mononuclear and neutrophils cells with abundant parasitic yeast form surrounded by collagen and reticuline fibers (Figure 1A) [10,23]. Moreover, using High-Resolution Computed Tomography (HRCT), nodular-diffuse, confluent and pseudo-tumoral lesions were observed, mainly located around the hilus, as well as peri-bronchial consolidations affecting more frequently the left lung of infected mice, findings that were histologically equivalent to a confluent and consolidated granulomatous reaction [26]. In the case of human patients with the chronic form of PCM, a study revealed that, at the moment of diagnosis, 93% of the patients showed infiltrative lesions with 31% of them presenting PF. It is noteworthy that 25% more of the studied patients developed PF at the end of the study; and PF correlated with the severity of infiltrates [7]. Moreover, in additional studies using HRCT and pulmonary functional analysis, it was described that inactive chronic PCM patients showed radiological abnormalities in 98% of cases being the most frequent the architectural distortion, followed by reticulate and septal thickening, centrilobular and paraseptal emphysema, and parenchymal bands, among others; however, despite these persistent radiological abnormalities, those patients showed short impairments in pulmonary functions and low impacts on aerobic capacity [27]. Noteworthy, there were not differences in the interstitial fibrotic tomographic abnormalities [27]; interestingly, those patients were treated with different antifungal drugs including itraconazole, sulfamethoxazole-trimethoprim, sulfadiazine, ketaconazole or a combination of two or more of these antifungals for treatment [27]. Furthermore, comparison of patients from two different geographic regions of Brazil, which were treated exclusively with cotrimoxazole, showed that the percentage of fibrosis was significantly higher in those evaluated patients from Botucatu (11.3%) than those from Campo Grande (2.3%); on the other hand, patients from Campo Grande presented more emphysema [28]. The above studies indicate that the presence of fibrosis not necessarily translate into severe functional deficits, a fact that could be related to the antifungal drug employed; additionally, the differences on clinical presentation, especially in the development of fibrosis or emphysema could be related to different causal cryptic species of the Paracoccidioides genus present in the geographic regions.

## 3. Therapeutic Approaches for Pulmonary Fibrosis in Paracoccidioidomycosis

Considering the complexity of the fibrotic process, the development of therapies has been challenging. Furthermore, therapeutic approaches that have proved successful in animal fibrosis models have failed in clinical trials, a fact that indicates important differences between humans and animal models. Thus, several compounds with anti-fibrotic or anti-inflammatory properties have been employed to treat IPF or bleomycin-induced PF at an experimental level; these compounds include pentoxifylline (PTX), azithromycin (AZT), and thalidomide (Thal), among others. PTX is characterized by its immunomodulatory properties; this compound has been shown to reduce the production of pro-inflammatory cytokines including TNF-α, IL-1-α, IL-6, and IL-8 [29], as well as to have exerted an anti-fibrotic effect through the inhibition of both fibroblast proliferation and ECM synthesis [30,31]. AZT, an antibiotic belonging to the macrolide group, has also been shown to exert an antifibrotic effect; thus, in a model of bleomycin-induced pulmonary fibrosis, AZT treatment reduces the production of both the pro-inflammatory cytokines IL-1β, IL-6, IL-17, and the chemokines MCP-1 and keratinocyte chemoattractant in the lungs [32]. Thal is recognized for its anti-inflammatory, immunomodulatory, and antiangiogenic activity [33,34,35,36,37]; Thal has been employed in an experimental model of bleomycin-induced PF, and the results have shown that Thal treatment reduces the deposition of type I collagen in the lungs [38,39]. Only in 2014, the U.S. Food and Drug Administration (FDA) approved two anti-fibrotic drugs, pirfenidone, and nintedanib, for the attenuation of IPF [40].

Nonetheless, there are currently no therapies available to reverse or treat PF in human PCM. In Table 1, the different experimental approaches that have been investigated for the treatment of PF induced in a pulmonary model of PCM are described.

### 3.1. Pharmacological Therapy

Currently, azole derivative drugs such as itraconazole (ITC) and voriconazole (VRC) as well as polyenes such as amphotericin B (AmB) are considered as the treatment of choice for endemic and systemic fungal infections including PCM, with ITC being the most widely used [44,45,46]. Moreover, the trimethoprim-sulfamethoxazole combination, also known as cotrimoxazole (CMX), has been largely employed and freely distributed in Brazil [46,47]. Nonetheless, these antifungal drugs show several disadvantages such as (*i*) azole compounds exert a fungistatic but not fungicidal effect against *P. brasiliensis* in vivo, (*ii*) although azoles are considered safe and efficacious, the duration of the treatment is long ranging from several months to a year depending on the patient’s condition, (*iii*) AmB is highly nephrotoxic, and (*iv*) these drugs do not attenuate PF [7,18].

Notably, it has been described that in addition to exerting an antifungal effect, ITC exhibits immunomodulatory properties [48]. Therefore, two studies in which the effect of ITC and PTX in a pulmonary model of PCM were carried out; in these studies, the effects of both ITC and PTX were evaluated independently as a monotherapy, and at histopathological and immunological levels. The authors observed that on starting the treatment of *P. brasiliensis*-infected mice with ITC or PTX at the early stages of infection (week four), reductions of the fungal burden, the granulomatous tissue inflammatory reaction, and fibrosis were observed [9,23]. In the case of ITC treatment, decreased levels of pro-inflammatory, and pro-fibrotic cytokines such as IL-1β, IL-13, TNF-α, and TGF-β [23] were also observed. On the other hand, *P. brasiliensis*-infected mice treated with PTX showed increased levels of granulocyte and macrophage colony-stimulating factor (GM-CSF), IL-12p70, IL-10, IL-13, and eotaxin, compared to untreated, infected-mice; moreover, the PTX treatment did not modify other pro-inflammatory cytokines [9]. Additionally, the same research group employed a combination of ITC plus PTX in the same model of *P. brasiliensis*-induced PF and found that the combined treatment used at advanced stages of infection (week eight) showed a reduction of the granulomatous inflammatory response and fibrosis as well as of the fungal burden; interestingly, there was only an increase in TGF-β levels, while the other pro-inflammatory cytokines remained unchanged compared to untreated or monotherapy-treated controls [8]. The above results suggest that prompt initiation of treatment would be necessary to avoid or reduce the development of fibrosis.

More recently, Finato et al. [12] evaluated the antifibrotic and antifungal combined therapies in an experimental model of pulmonary PCM. Thus, these authors investigated the antifibrotic activity of PTX, AZT, and Thal in combination with the antifungals ITC or CMX in *P. brasiliensis*-infected mice at advanced stages of infection (week eight). Of note, the authors confirmed the antifibrotic effect exerted by the combination ITC + PTX as previously reported by Naranjo et al. [8]; thus, those treated infected mice showed a reduction in the pulmonary concentration of hydroxyproline associated with lower concentrations of IL-6, IL-17, and TGF-β and higher concentrations of IL-10. Additionally, infected mice treated with a combination of CMX + AZT also exhibited low levels of hydroxyproline and TGF-β with higher levels of IL-10. On the contrary, the combined treatment with ITC + Thal, CMX + Thal, and ITC + AZT was associated with the loss of body weight, the increased deposition of reticuline fibers, low levels of IL-1β, IL-6, and TGF-β, and higher concentrations of vascular endothelial growth factor (VEGF), interferon-gamma (IFN-γ), and CCL3 [12].

Finally, in another recent study, a new compound, CP1, with an antifungal effect, was evaluated [41]. CP1 inhibits the enzymatic activity of the chorismate synthase from *P. brasiliensis*. This enzyme takes part in the shikimate pathway and is responsible for the synthesis of chorismite, which, in turn, participates in the biosynthesis of several important aromatic molecules, including aromatic amino acids, folate, naphthoquinones, and menaquinones [49]. A pulmonary PCM model that developed an early PF at two weeks after infection was employed to evaluate the CP1 effect. This new antifungal compound was used at the beginning of infection (two days post-infection) and daily for two weeks, and then the lungs of the mice were analyzed for fungal burden and histopathological analysis; the results showed a reduction not only of the fungal burden but also in the pulmonary inflammatory response and efficiently protected against PF [41].

### 3.2. Immunotherapy or Antibody-Based Therapy

As previously mentioned, treatment for fibrosis represents a challenge because currently there is not an effective therapy to counteract the fibrotic process. Thus, there is an urgent need for new, more effective, and well-tolerated therapies for PF. Accordingly, immunotherapy has provided a breakthrough in several autoimmune diseases; however, it is associated with an increased risk of infections. In the last decade, antibody-based therapies with specific targets have been investigated at length in IPF as an alternative or complementary treatment aiming to ameliorate the relentless fibrotic process of IPF [50], as well as in PF in radiation- and bleomycin-induced models. Several monoclonal antibodies (mAbs) addressed against known fibrogenic factors and matrix components including laminin receptor-1 [51], Krebs von den Lungen (KL)-6 [52], and connective tissue growth factor (CTGF) [53]; as well as those developed to antagonize the inflammation and immunity pathways such as IL-13 [54]; TNF-α [55]; TGF-β [56]; platelet-derived growth factor (PDGF) [57]; HER2, a membrane-bound protein belonging to the epidermal growth factor receptor family (EGFR) [58]; follistatin-like 1, a TGF-β inducible gene [59]; CCL24 [60]; CXCL6 [61]; CXCR4 [62]; OX40L, a T cell costimulatory signal molecule [63]; and CD25 (Treg cells) [64], among several other targeted molecules, have been evaluated. All these specific mAbs successfully reduced or alleviated PF. Additionally, some of these mAbs have been evaluated in phase II clinical trials such as Pamrevlumab (specific to CTGF) [53] and Tralokinumab (specific to IL-13) [65].

Regarding PF due to *P. brasiliensis* infection, a strategy using a mAb specific to neutrophils was employed [10,11]. It has been described that neutrophils play an important role in the pathogenesis of PCM; thus, these phagocytic cells exert a protecting effect during the early stages of infection [66], as well as being relevant during the chronic stage of the mycosis as they are present in high numbers accompanying the granulomatous lesions [8,23,67]. With this in mind, *P. brasiliensis*-infected mice were treated with a mAb specific to neutrophils at four weeks post-infection (when the fibrotic process begins) followed by doses every two days for two weeks. Histopathological and immunological analyses were then carried out after four and eight weeks. The results of this study showed that depletion of neutrophils using the specific mAb was correlated with a reduction in the number of pro-inflammatory cells (eosinophils, CD4 T- and B- cells, MDSCs, Treg), fungal load, and pro-inflammatory cytokines including IL-17, TNF-α, and TGF-β1. Moreover, this immunotherapy showed an important reduction in the expression of pro-fibrotic molecules (collagen, TGF-β3, MMP-12, MMP-14) and an increase of anti-fibrotic ones (MMP-8 and TIMP-2), which, in turn, was associated with an attenuation of inflammation and lung fibrosis, as well as a lower deposition of collagen and reticulin fibers with a recovery of the lung architecture (Figure 1) [10]. Subsequently, the effect of ITC in combination with the mAb anti-neutrophils was also employed in the PCM model [11]. In this study, it was observed that the combination of ITC + mAb favored the control of infection and reduced the inflammatory response and PF. Notably, this therapeutic strategy, therefore, reduced the expression of several inflammatory and pro-fibrotic (IL-1β, IL-6, IL-17, IL-10, TNF-α, TGF-β1, GATA-3, RORc, Ahr, MMP-1α, MMP-15, TIMP-1, and TIMP-2) genes in an additive manner compared with those animals that received monotherapy [11]. The above findings suggest that the implementation of immunotherapy strategies targeting specific molecules or cells could be effective in the treatment of PF in PCM.

### 3.3. Cellular Therapy

Despite new experimental approaches using pharmacological and immunotherapy strategies to ameliorate IPF and bleomycin-induced PF models having been shown to be effective, the damaged lung tissue does not recover at all. Thus, there is a need to establish regenerative therapies. Accordingly, cell-based therapies have shown great potential to treat lung diseases, and it has been proposed that the administration of cells into injured lungs could be considered as a good therapeutic approach to repair and replace damaged or lost lung tissue [68]. Among these cell-based therapies, stem cell-based approaches include the use of mesenchymal stem cells (MSCs), which have shown to lead to an improvement in bleomycin-induced collagen deposition in animal lungs and PF [69]. MSCs exhibit several properties; thus, they can: (*i*) migrate at sites of injury, (*ii*) modulate the immune responses, (*iii*) repair epithelial tissues, (*iv*) attenuate extracellular matrix deposition, and (*v*) modify the microenvironment at the engraftment sites, as well exhibit antiapoptotic properties [70,71]. Different approaches using MSC-based therapy for treating PF have been reported; these include bone marrow MSCs (BMMSCs) [72], lung spheroid cells [73], human umbilical MSCs (HUMSCs) from Wharton’s jelly [74], preconditioned MSCs [75], and induced pluripotent stem (iPS) cells [76]. The results of the above studies showed that the transplantation of these MSCs was able to prevent or reduce fibrosis.

**Figure 2:**
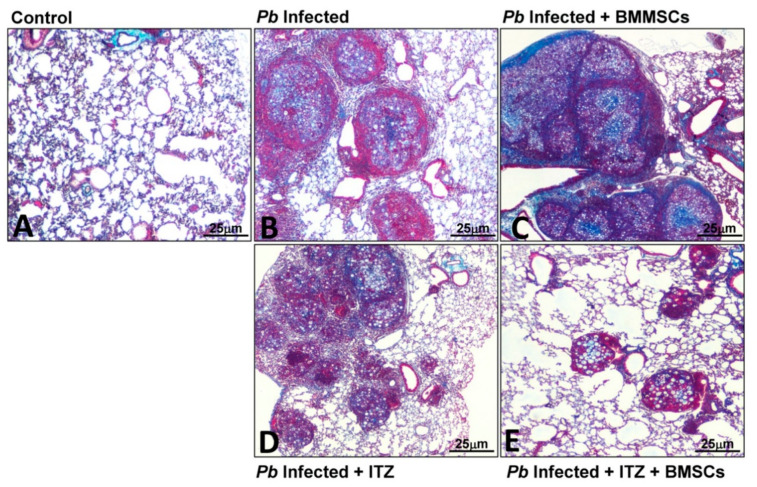
Combined therapy using itraconazole and bone marrow mesenchymal stem cells (BMMSCs) reduces pulmonary inflammation and fibrosis in the lungs of mice infected with *P. brasiliensis*. Microphotographs represent the lungs from (**A**) uninfected mice; (**B**) mice infected with *P. brasiliensis*; (**C**) infected and BMMSCs-treated animals at week eight post-challenge; (**D**) infected and Itraconazole (ITC) treated-mice at week six post-infection, and (**E**) lungs from infected mice treated with ITC at 6th-week p.i. and transplanted with BM-MSCs at week eight post-challenge. The lungs were fixed in formalin, embedded in paraffin, cut, and stained with Masson’s trichrome stain to determine injured and fibrotic lungs areas. Magnification 10×.

Concerning PF in PCM, a cell-based therapy approach using BMMSCs has been employed. Contrary to what was expected, transplantation of BMMSCs in *P. brasiliensis*-infected mice at eight weeks (when PF has been established) exacerbated not only the course of the disease but also PF. Thus, four weeks after BMMSCs transplantation, an increase in fungal burden, inflammatory cells (including neutrophils, eosinophils, and M2 macrophages), fibrocytes, proinflammatory cytokines and chemokines (IL-6, IL-9, GM-CSF, CXCL1, CXCL9, and CCL5), soluble collagen, and pro-fibrotic genes expression (collagen-3α1, TGF-β3, and MMP-15) were observed. The lungs also showed an increased inflammatory process with fibrosis [24,42]. It was hypothesized that this outcome could be triggered by either the inflammatory microenvironment induced during the disease or by interaction with the fungus. Thus, human fibroblasts were stimulated with homogenized lung supernatants from infected and BMMSCs-transplanted mice, which showed a higher expression of collagen I [24]. More recently, while performing in vitro experimental studies, it was demonstrated that the above results were also due, in part, to a direct interaction between *P. brasiliensis* and BMMSCs. In this study, it was observed that fungal cells activate BMMSCs through a mechanism dependent on *Toll*-Like receptors (TLR)-2, TLR4, and Dectin-1, and trigger the expression of inflammatory mediators such as IL-6, IL-17, TNF-α, and TGF-β [77]. Interestingly, in those *P. brasiliensis*-infected mice transplanted with BMMSCs and previously treated with ITC, it was observed that the combined treatment induced a synergistic reduction of Col3α1, TGF-β3, MMP-8, MMP-12, and TIMP-1, as well as an increase of TIMP-2 gene expression compared with infected mice that received cell transplantation; additionally, lung histopathological analysis of mice that received the combined therapy showed a marked reduction in the inflammatory response and fibrosis in comparison with those mice that only received either monotherapy (Figure 2) [24]. These results suggest that the late transplantation of BMMSCs in the PCM model does not have any anti-fibrotic effect; however, combined treatment strategies shed light on the use of cell-based therapy, especially when used in combination with antifungal therapy.

### 3.4. Vaccination

Immunization based on recombinant proteins has been widely used to prevent the development of infectious diseases. However, this strategy has not been evaluated to avoid, or prevent, the development of sequelae such as fibrosis induced by infectious agents.

Accordingly, it has been previously shown that protein-based vaccination using a 27-kDa recombinant protein (rPb27) of *P. brasiliensis* provides protection against PCM [78,79]. Subsequently, the development of PF was evaluated in mice immunized with the rPb27; in this study, the immunized mice showed a lower level of fibrosis as determined by histology and a reduced expression of collagen. Additionally, those immunized mice showed high levels of IFN-γ, TGF-β, and IL-10; as well as high activity of caspase 3, an enzyme associated with protection against exacerbated inflammatory responses; and reduced levels of CCR7, a chemokine receptor expressed on fibroblast and involved in the PF development [43]. This study suggests that the mechanism exerted by rPb27 vaccination against fibrosis development induced by *P. brasiliensis* is through an early reduction in the fungal load, thus avoiding the persistent fungal antigen stimulations and the subsequent activation of the immune system.

## 4. Conclusions and Future Directions

Over the last decade, several studies have evaluated different therapeutic approaches for treating the main sequelae developed in chronic PCM, pulmonary fibrosis, which develops in almost 60% of patients suffering the mycosis even after treatment with antifungal therapy for long periods. Although the main developments for the treatment of fibrosis obtained so far have been focused on fibrosis due to other agents different from those infectious or microbial, such strategies are of great value because they serve as models for infectious agents induced-PF. Altogether, the studies reported above that focused on the treatment of PF in PCM suggest that the combined therapies appear to be more effective; thus, the uses of immunomodulatory agents including PTX and AZT, as well as the use of immunotherapy, especially monoclonal antibodies, and cell-based therapy using MSCs, all of them combined with antifungals appear to be promising and successful strategies to treat these sequelae. However, it is important to consider that the use of cell-based therapies to treat PF is progressing and is still at the experimental phase; therefore, some pitfalls that should be resolve include the safety of cell transplantation, routes of delivery, and the dose and timing of administration, as well as its efficacy, should be evaluated, especially when used in combination with pharmacological agents. Additionally, future studies will need to evaluate immunotherapy-based strategies in PF due to *P. brasiliensis* infection, especially to assess mAbs against new targets. Prophylactic vaccination using new antigens, as well as new antifungal compounds, should also be considered.

Overall, these findings open the door to implementing new therapy strategies using antimicrobials in combination with new biological agents or cell-based therapies capable of enhancing or modulating the immune response. Nonetheless, it is time to consider moving these studies to clinical trials, especially those that have used antifungal and other immunomodulatory agents authorized for use in humans.

## Figures and Tables

**Table 1:** Experimental therapy strategies employed in mouse models of pulmonary fibrosis induced by *Paracoccidioides brasiliensis* infection.

Therapy Approach	Findings	References
Drugs		
ITC	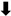 Fungal burden, inflammatory response; and PF if treatment was started an early time post-infection (4 wk.) 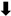 IL-1β, IL-13, TNF-α and TGF-β	[23]
PTX	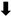 Fungal burden, inflammatory response; and PF if treatment was started an early time post-infection (4 wk.) 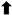 GM-CSF, IL-12p70, IL-10, IL-13 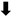 RANTES	[9]
ITC + PTX	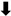 Fungal burden, inflammatory response; and PF even if treatment was started an advanced time post-infection (8 wk.) 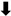 Hydroxyproline 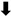 IL-1β; IL-6, IL-17, TGF-β1 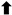 IL-10	[8,12]
ITC + AZT	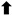 PF, CCL3, IL-17, IFN-γ, VEGF 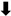 IL-1β, IL-6, IL-10, TGF-β1	[12]
ITC + Thal	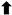 PF 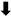 IL-1β, IL-6, IL-10, IL-12, IL-17, TGF-β1, VEGF, IFN-γ, CCL3	[12]
CMX + PTX	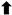 PF, CCL3 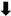 IL-17, TGF-β1	[12]
CMX + AZT	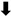 Inflammatory response, hydroxyproline 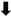 TGF-β1 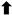 TNF-α, IL-10	[12]
CMX + Thal	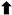 PF 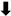 IL-1β, IL-6, IL-17, TGF-β1, VEGF, IFN-γ, CCL3	[12]
CP1	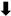 Fungal burden, Inflammatory response, PF	[41]
Biological		
mAbs		
anti-neutrophils	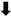 Fungal burden, Inflammatory response, PF 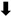 IL-17, TNF-α, TGF-β1, TGF-β3, MMP-12, MMP-14 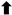 MMP8, TIMP-2	[10]
anti-neutrophil + ITC	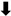 Fungal burden, Inflammatory response, PF 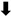 IL-1β, IL-6, IL-17, IL-10, TNF-α, TGF-β1, MMP-1α, 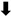 GATA-3, RORc, Ahr, 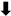 MMP-15, TIMP-1, TIMP-2	[11]
MSCs		
BMMSCs	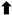 Fungal burden, inflammatory response, PF 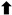 IL-6, IL-9, GM-CSF, CXCL1, CXCL9, CCL5 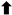 Collagen-3α1, TGF-β3, MMP-15	[24,42]
BMMSCs + ITC	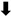 Fungal burden, inflammatory response, PF 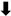 Col3α1, TGF-β3, MMP-8, MMP-12, TIMP-1 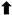 TIMP-2	[24]
Vaccines		
rPb27	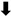 PF, collagen 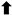 IFN-γ, TGF-β, IL-10 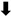 CCR7 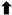 Activity of caspase 3	[43]

PF, pulmonary fibrosis; ITC, itraconazole; PTX, pentoxifylline; AZT, azithromycin; Thal, thalidomide; CMX, cotrimoxazole; CP1, an antifungal compound that inhibits the enzymatic activity of the chorismate synthase; mAbs, monoclonal antibodies; MSCs, mesenchymal stem cells; BMMSCs, bone marrow MSCs; rPb27, recombinant protein of 27 kDa of *Paracoccidioides brasiliensis*; TNF-α, tumor necrosis factor-alpha; transforming growth factor-beta, TGF-β; GM-CSF, granulocyte and macrophage colony-stimulating factor; RANTES, regulated upon activation, normal T cell expressed and secreted; VEGF, vascular endothelial growth factor; MMP, metalloproteinase; TIMP, tissue inhibitor metalloproteinase; wk., week.
